# DCDC2 inhibits hepatic stellate cell activation and ameliorates CCl_4_-induced liver fibrosis by suppressing Wnt/β-catenin signaling

**DOI:** 10.1038/s41598-024-59698-w

**Published:** 2024-04-24

**Authors:** Qing-Qing Liu, Jing Chen, Tao Ma, Wei Huang, Cui-Hua Lu

**Affiliations:** 1 Suzhou Medical College of Soochow University, Suzhou, 215000 China; 2 Department of Gastroenterology, Affiliated Hospital of Nantong University, Medical School of Nantong University, Nantong, 226001 China

**Keywords:** DCDC2, Hepatic stellate cell activation, Liver fibrosis, Wnt/β-catenin signaling, Cell biology, Molecular biology, Liver fibrosis

## Abstract

Liver fibrosis, as a consequence of chronic liver disease, involves the activation of hepatic stellate cell (HSC) caused by various chronic liver injuries. Emerging evidence suggests that activation of HSC during an inflammatory state can lead to abnormal accumulation of extracellular matrix (ECM). Investigating novel strategies to inhibit HSC activation and proliferation holds significant importance for the treatment of liver fibrosis. As a member of the doublecortin domain-containing family, doublecortin domain containing 2 (DCDC2) mutations can lead to neonatal sclerosing cholangitis, but its involvement in liver fibrosis remains unclear. Therefore, this study aims to elucidate the role of DCDC2 in liver fibrosis. Our findings revealed a reduction in DCDC2 expression in both human fibrotic liver tissues and carbon tetrachloride (CCl_4_)-induced mouse liver fibrotic tissues. Furthermore, exposure to transforming growth factor beta-1(TGF-β1) stimulation resulted in a dose- and time-dependent decrease in DCDC2 expression. The overexpression of DCDC2 inhibited the expression of α-smooth muscle actin (α-SMA) and type I collagen alpha 1 (Col1α1), and reduced the activation of HSC stimulated with TGF-β1. Additionally, we provided evidence that the Wnt/β-catenin signaling pathway was involved in this process, wherein DCDC2 was observed to inhibit β-catenin activation, thereby preventing its nuclear translocation. Furthermore, our findings demonstrated that DCDC2 could attenuate the proliferation and epithelial-mesenchymal transition (EMT)-like processes of HSC. In vivo*,* exogenous DCDC2 could ameliorate CCl_4_-induced liver fibrosis. In summary, DCDC2 was remarkably downregulated in liver fibrotic tissues of both humans and mice, as well as in TGF-β1-activated HSC. DCDC2 inhibited the activation of HSC induced by TGF-β1 in vitro and fibrogenic changes in vivo, suggesting that it is a promising therapeutic target for liver fibrosis and warrants further investigation in clinical practice.

## Introduction

Liver fibrosis is a common feature of chronic liver diseases, primarily arising from recurring liver injuries caused by various factors, including viral hepatitis infections, alcohol consumption, non-alcoholic steatohepatitis, or cholestasis^1,2^. The excessive deposition and abnormal distribution of extracellular matrix (ECM) components, such as collagen, glycoproteins, and proteoglycans within the liver, signifies the pathological repair response of the liver to chronic injuries^3–5^. This process serves as a crucial stage in the progresion of various chronic liver diseases towards cirrhosis and significantly influences their prognosis^6^. Liver fibrosis is histologically reversible and can be effectively reversed with aggressive treatment during this phase. However, if left untreated, fibrosis may advance to cirrhosis, wherein reversal becomes markedly challenging, leading to a relatively poor prognosis^7–9^. Globally, liver fibrosis/cirrhosis poses a significant health burden, contributing to liver failure or hepatocellular carcinoma (HCC), and resulting in numerous deaths annually^10,11^. The pathogenesis of liver fibrosis/cirrhosis is highly intricate, presenting significant challenges in treatment^12,13^. Therefore, research on liver fibrosis has consistently remained a focal point in liver disease studies.

It is widely acknowledged that hepatic stellate cell (HSC) remain quiescent under normal conditions. However, in response to liver injuries such as inflammation or mechanical stimuli, HSC become activated, transitioning from a quiescent to an activated phenotype. During this activation process, HSC acquire a myofibroblast-like phenotype, transdifferentiating into myofibroblasts, and participate in liver fibrosis formation by proliferating and secreting ECM components, thus increasing the expression of α-smooth muscle actin (α-SMA) and type I collagen alpha 1 (Col1α1). These processes play a crucial role in the development of liver fibrosis^14–16^. Among various cytokines, transforming growth factor beta-1 (TGF-β1) stands out as the most potent one, exerting an important role in the activation of HSC^17,18^. Dysregulation of numerous signaling pathways and gene expression patterns occurs during this process. Particularly, the Wnt/β-catenin signaling pathway plays a pivotal role in mediating the progression of liver fibrosis^19,20^. The Wnt/β-catenin pathway is important for regulating cell growth and proliferation, and it holds great significance in maintaining normal liver growth and function^21^. Inhibition of the β-catenin signaling pathway, leads to a decrease in the activation and proliferation of HSC, thereby attenuating liver fibrosis^22–24^. Therefore, gaining deeper insights into the connection between the activation of HSC and the Wnt/β-catenin signaling pathway is crucial for developing effective treatments for liver fibrosis.

The doublecortin domain containing 2 (DCDC2), encoded in humans by the *DCDC2* gene, is located at 6p22.1^25^. The protein encoded by this gene contains two microtubule-associated protein peptide domains known to bind to tubulin, thereby facilitating microtubule polymerization. This gene mutation is originally linked to dyslexia^26^. Recent reports have reported and confirmed a correlation between DCDC2 and the occurrence and development of tumors. Notably, it has been confirmed that DCDC2 can inhibit the progression of HCC^27^. Additionally, high-throughput exon sequencing has identified a recessive truncated mutation of *DCDC2* in patients with a renal-hepatic ciliopathy and subsequent investigations have revealed that such patients often exhibit complications with liver fibrosis, cirrhosis, and even liver cancer at an early stage^28^. However, to date, no studies have explored the potential relationship between DCDC2 and liver fibrosis.

To our knowledge, this is the first study to demonstrate the important role of DCDC2 in the pathogenesis of liver fibrosis and determine its potential underlying mechanism. Our initial findings revealed a significant down-regulation of *DCDC2* expression, both at the protein and mRNA levels, in human and mouse liver fibrosis models compared to their respective normal counterparts. Furthermore, we confirmed that the overexpression of *DCDC2* effectively inhibited the activation of HSC and the expression of β-catenin, thereby reducing its nuclear translocation. This ultimately suppressed downstream gene expressions and inhibited cell proliferation. Previous evidence has suggested that epithelial-mesenchymal transition (EMT) plays a crucial role in liver fibrosis and is closely associated with Wnt/β-catenin signaling^29,30^. Extensive research has provided compelling evidence indicating that HSC possess the ability to accelerate their transition into myofibroblasts through the process of EMT, thereby leading to an enhanced proliferation of HSC^31^. This activity leads to a significant increase in the activation of HSC and the accumulation of ECM in the liver, exacerbating the progression of liver fibrosis^32^. When describing this process in HSC, we use the term “EMT-like transition” to distinguish it from traditional EMT, considering their liver-specific mesenchymal nature. Subsequent investigations revealed that the enforced expression of DCDC2 inhibited the expression of mesenchymal markers (such as N-cadherin, vimentin, and Snail) while increasing the expression of epithelial markers (such as E-cadherin). Finally, we demonstrated that injecting lentivirus into the tail vein, which delivered DCDC2 expression to the liver, effectively improved liver fibrosis. This improvement was mediated by regulating the Wnt/β-catenin signaling pathway. In conclusion, our objective was to identify a key factor in HSC activation, thereby establishing a robust experimental foundation for understanding its role in liver fibrosis.

## Results

### Low expression of DCDC2 in human and mouse liver fibrotic tissues

**Figure 1:**
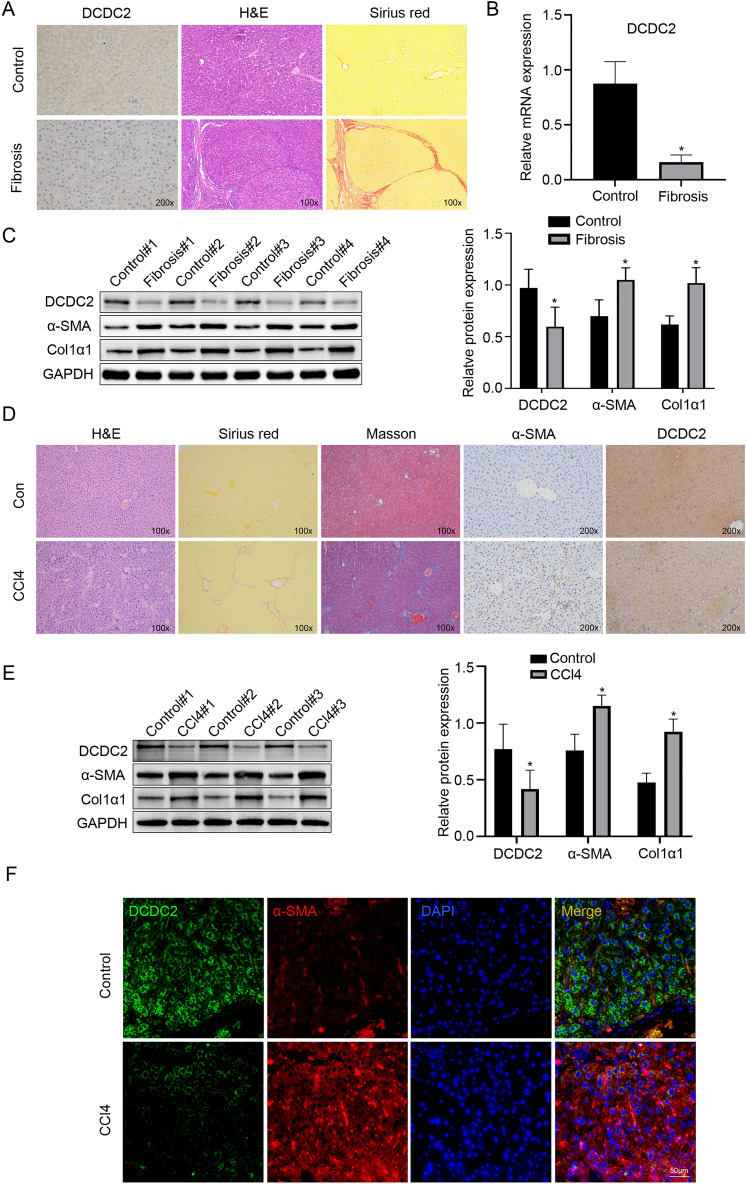
Low expression of DCDC2 in human and mouse liver fibrotic tissues. (**A**) Hematoxylin and eosin (H&E), Sirius red staining and immunohistochemistry of DCDC2 were performed in tissues from human healthy controls and liver fibrosis samples. (**B**) The mRNA expression level of DCDC2 was measured using qPCR in human healthy control and liver fibrosis samples. (**C**) The protein expression levels of DCDC2, α-smooth muscle actin (α-SMA), and type I collagen alpha 1 (Col1α1) were assessed via western blot analysis in human healthy control and liver fibrosis samples. (**D**) H&E, Sirius red staining, Masson trichrome staining and immunohistochemistry analyses were conducted on liver tissues obtained from mice. (**E**) The protein expression levels of DCDC2, α-SMA, and Col1α1 were assessed via western blot analysis in liver tissues obtained from mice. (**F**) Immunofluorescence staining of DCDC2 (green) and α-SMA (red) in liver tissues from mice. Scale bar, 50 µM. The experiment was repeated at least three times and statistical data were presented as mean ± SD. * compare with Control, * *P* < 0.05.

To investigate the expression levels of DCDC2 in liver fibrosis, we obtained tissue samples from healthy individuals as well as those with liver fibrosis. Immunochemistry analysis revealed that patients with liver fibrosis exhibited decreased tissue expression of DCDC2 compared to the control groups (Fig. 1A). To verify these findings, we conducted further assessments of DCDC2 changes utilizing western blot and quantitative real-time polymerase chain reaction (qPCR). The levels of DCDC2 mRNA and protein were significantly reduced in the liver fibrosis groups (Fig. 1B,C). Conversely, the expression of α-SMA and Col1α1 was notably elevated in liver fibrotic tissues (Fig. 1B,C). Subsequently, we successfully established a mouse model of liver fibrosis via intraperitoneal injection with carbon tetrachloride (CCl_4_). Compared to the control groups, a significant increase in the staining for α-SMA and Col1α1 was observed, whereas the staining for DCDC2 was markedly reduced (Fig. 1D). The western blot results corroborated these findings (Fig. 1E). Furthermore, we conducted a study to determine the co-localization of DCDC2 and α-SMA through immunofluorescence double staining in vivo. Interestingly, the outcome revealed a prominent downregulated of DCDC2 expression in activated HSC (α-SMA positive) (Fig. 1F). These results suggested that DCDC2 expression was diminished in liver fibrosis and was linked to the activation of HSC.

### Downregulation of DCDC2 in HSC activated by TGF-β1

**Figure 2:**
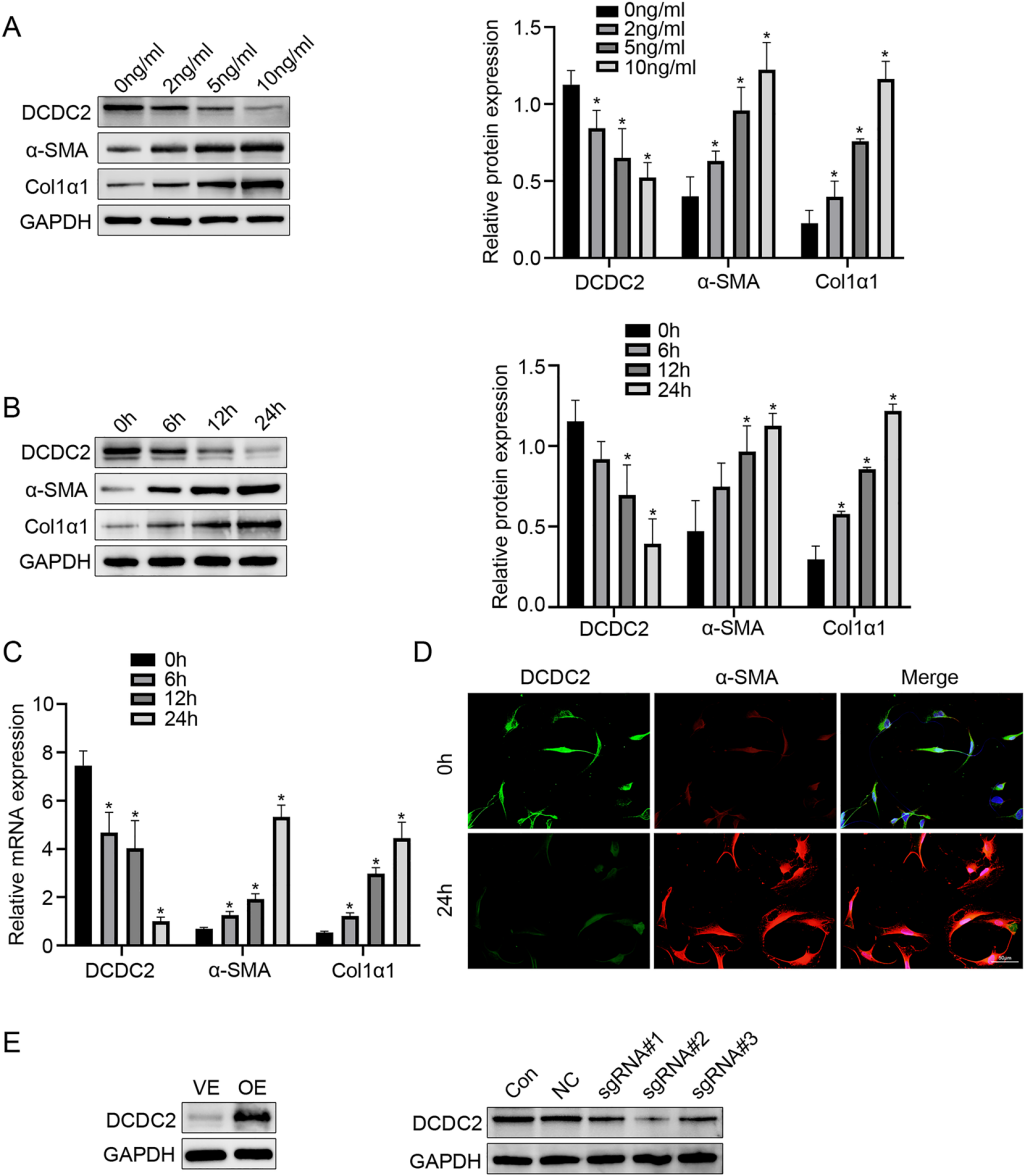
Downregulation of DCDC2 in HSC activated by TGF-β1. (**A**) LX-2 cells were treated with different concentrations of TGF-β1 for 24 h. The protein expression levels of α-SMA and Col1α1 were detected using western blot. (**B**) LX-2 cells were treated with 10 ng/mL TGF-β1 at different time points. The protein expression levels of DCDC2, α-SMA and Col1α1 were assessed using western blot analysis. (**C**) The mRNA levels of DCDC2, α-SMA and Col1α1 were measured using qPCR. (**D**) Immunofluorescence staining of DCDC2 (green) and α-SMA (red) in LX-2 cells at 0 and 24 h following treatment with 10 ng/mL TGF-β1. (**E**) The efficiency of DCDC2 overexpression or silencing was measured by western blot. Scale bar, 50 µM. The experiment was repeated at least three times and statistical data were presented as mean ± SD. * compare with TGF-β1 0 ng/mL group (A) or TGF-β1 10 ng/mL 0 h group (D), * *P* < 0.05.

To further investigate the relationship between DCDC2 and HSC activation, we established an in vitro model of TGF-β1-induced HSC activation. LX-2 cells were stimulated with varying concentrations of recombinant TGF-β1 for 24 h to induce HSC activation. Following treatment, we observed a dose-dependent increase in the expression of α-SMA and Col1α1, while the expression of DCDC2 decreased (Fig. 2A). Owing to the highest inhibition efficiency at a concentration of 10 ng/mL, we chose it for subsequent studies. LX-2 cells were stimulated with 10 ng/mL and harvested at various time points. We observed a time-dependent increase in α-SMA and Col1α1 expression levels in LX-2 cells stimulated with TGF-β1. In contrast, the expression of DCDC2 decreased over time (Fig. 2B,C). Additionally, immunofluorescence staining revealed reduced DCDC2 expression and increased α-SMA expression in LX-2 cells stimulated with TGF-β1 (Fig. 2D). Following transfection of LX-2 cells with lentiviral vector or single guide RNA (sgRNA) carrying the DCDC2 target gene, the efficiency was assessed using western blot analysis (Fig. 2E).

### DCDC2 inhibits the activation of HSC induced by TGF-β1 by targeting the Wnt/β-catenin pathway

**Figure 3:**
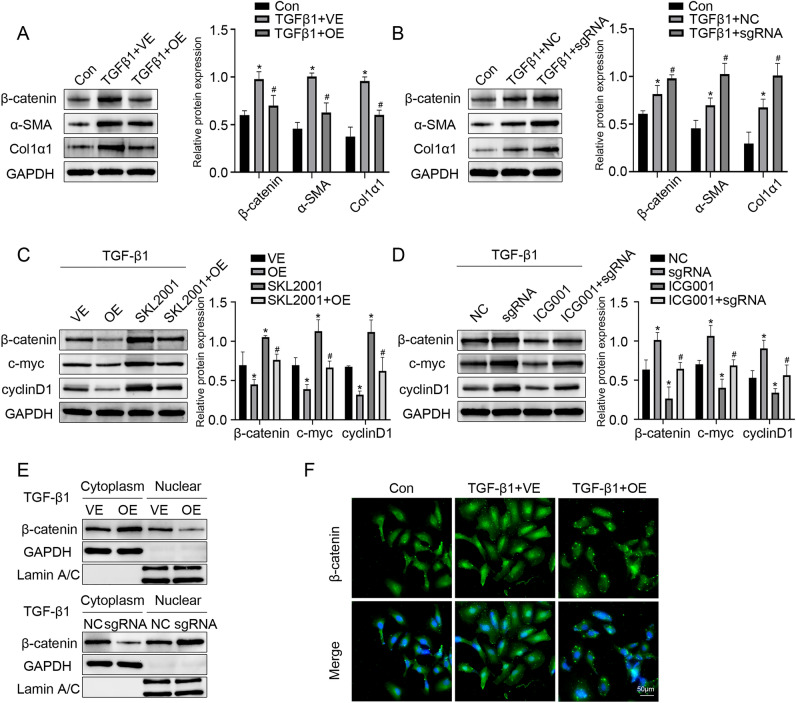
DCDC2 inhibits the activation of HSC induced by TGF-β1 by targeting the Wnt/β-catenin pathway. (**A**) The protein expression levels of β-catenin, α-SMA, and Col1α1 were assessed via western blot analysis subsequently to transfecting LX-2 cells with DCDC2 overexpressing lentiviral vector and negative control vector, followed by stimulation with TGF-β1. (**B**) The protein expression levels of β-catenin, α-SMA, and Col1α1 were evaluated through western blot analysis after transfecting LX-2 cells with DCDC2-sgRNA and negative control sgRNA, followed by stimulation by TGF-β1. (**C**) The protein expression levels of β-catenin, cyclinD1, and c-myc were detected by western blot after LX-2 cells were transfected with DCDC2 overexpressing lentiviral vector and/or then stimulated by SKL2001. (**D**) The protein expression levels of β-catenin, cyclinD1, and c-myc were assessed via western blot analysis after transfecting LX-2 cells with DCDC2-sgRNA and/or then stimulated by ICG001. (**E**) The protein expression levels of β-catenin in the cytoplasm or nuclear after DCDC2 was overexpressed or knockdown was measured using western blot. (**F**) Immunofluorescence staining of β-catenin nuclear translocation in LX-2 cells following DCDC2 was overexpressed. Scale bar, 50 µM. The experiment was repeated at least three times and statistical data were presented as mean ± SD. * compare with Con, # compare with TGF-β1 +VE group (A, B), OE group (C) or sgRNA group (D), *, # *P* < 0.05.

To discuss the effect of DCDC2 on HSC activation, LX-2 cells were transfected with DCDC2-overexpressing plasmid, and then incubated with TGF-β1 for 24 h. The expression of α-SMA and Col1α1 significantly enhanced in activated HSC, which were reversed following the overexpression of DCDC2 (Fig. 3A). Previous studies have confirmed that the Wnt/β-catenin signaling pathway plays a crucial role in liver fibrosis. Compared to the TGF-β1 treatment group, reduced expression of β-catenin was observed in the DCDC2 overexpression group (Fig. 3A). Subsequently, to validate these findings, DCDC2 sgRNA was transfected into LX-2 cells, resulting in a marked increase in β-catenin expression, consistent with expectations (Fig. 3B). To further investigate the effect of DCDC2 on the Wnt/β-catenin signaling pathway, we used SKL2001 (a β-catenin agonist) and ICG-001 (a β-catenin inhibitor). The obvious reduction of β-catenin induced by the overexpression of DCDC2 was notably restored by SKL2001 (Fig. 3C). Similarly, the markedly increased levels of β-catenin following DCDC2-sgRNA transfection were partially reversed (Fig. 3D). As it is well known, nuclear accumulation of β-catenin is pivotal for pathway to activation. Additionally, we observed a reduction in nuclear β-catenin due to the overexpression of DCDC2, with a concomitant increase in the cytoplasm (Fig. 3E). Consistent with this observation, immunofluorescence staining confirmed that nuclear translocation of β-catenin in TGF-β1-treated LX-2 cells was attenuated by DCDC2 overexpression (Fig. 3F). These findings collectively suggested that DCDC2 potentially inhibits the nuclear translocation of β-catenin to suppress its activity, implicating the involvement of the Wnt/β-catenin signaling pathway in DCDC2’s inhibitory effect on HSC activation.

### DCDC2 suppresses HSC proliferation and attenuates EMT-like transitions in vitro

**Figure 4:**
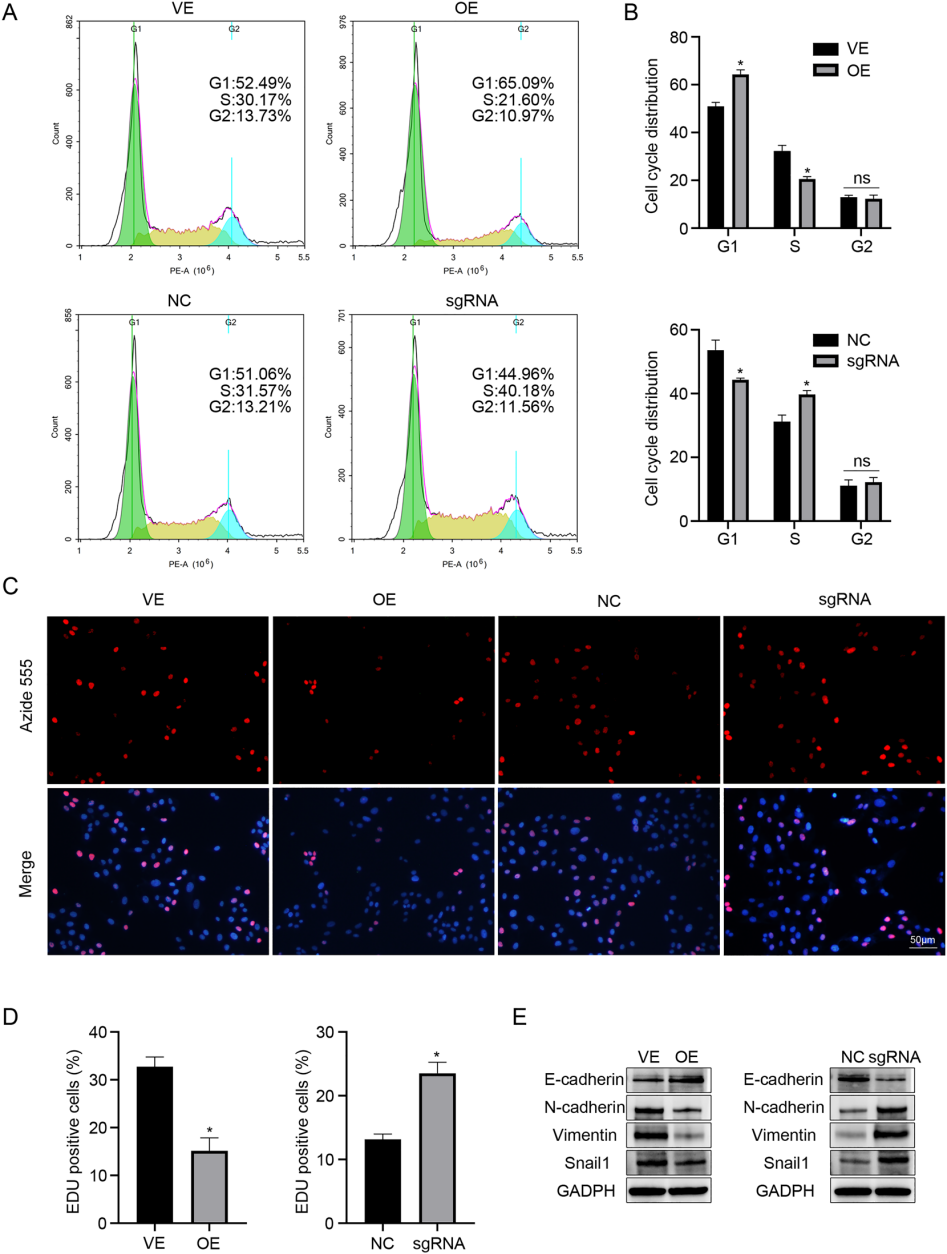
DCDC2 suppresses HSC proliferation and attenuates EMT-like transitions in vitro. (**A**,**B**) The cell cycle phase distribution was measured using flow cytometry analyses after LX-2 cells were transfected with DCDC2 overexpressing lentiviral vector or DCDC2-sgRNA. (**C**,**D**) EdU assay was performed to examine the proliferation of LX-2 cells following DCDC2 overexpression or knockdown. (**E**) The protein expression of E-cadherin, N-cadherin, vimentin, and Snail1 was detected using western blot after LX-2 cells were transfected with DCDC2 overexpressing lentiviral vector or DCDC2-sgRNA. The experiment was repeated at least three times and statistical data were presented as mean ± SD. * compare with VE group or NC group, *P < 0.05.

Following DCDC2 overexpression, we observed a decrease in the levels of cyclinD1 and c-myc, both of which are downstream target genes of β-catenin (Fig. 3C). Numerous studies have confirmed that cyclin D1 and c-myc are involved in regulating the cell cycle and cell proliferation. To further validate the effect of DCDC2 on the cell proliferation of HSC, we assessed the cell cycle phase distribution through flow cytometry analyses. The results revealed an increase in the G0/G1 phase and a decrease in the S phase in the LX-2 cells following treatment with DCDC2 overexpression. Conversely, a decrease in the G0/G1 phase and an increase in the S phase were observed in LX-2 cells following DCDC2 knockdown (Fig. 4A). Furthermore, EdU assays demonstrated a negative effect of DCDC2 on the cell proliferation of HSC (Fig. 4B). In summary, DCDC2 suppressed HSC proliferation by inducing cell cycle arrest at the G0/G1 phase. Existing research findings have indicated that EMT plays a crucial role in the process of liver fibrosis. Moreover, increasing evidence suggests that HSC may undergo epithelial-mesenchymal/mesenchymal-epithelial-like transitions and exhibit expression of both epithelial and mesenchymal genes^33^. Hence, we sought to assess the expression of EMT-related genes in LX-2 cells with DCDC2 overexpression or knockdown. Following DCDC2 overexpression, E-cadherin levels markedly increased, while N-cadherin, vimentin, and Snail levels decreased (Fig. 4C). Conversely, E-cadherin levels significantly decreased, whereas N-cadherin, vimentin, and Snail levels increased with DCDC2 knockdown (Fig. 4D). These findings demonstrated that DCDC2 inhibited the proliferation of HSC and mitigated EMT-like transitions.

### Overexpression of DCDC2 improves liver fibrosis in CCl_4_-induced mouse model

**Figure 5:**
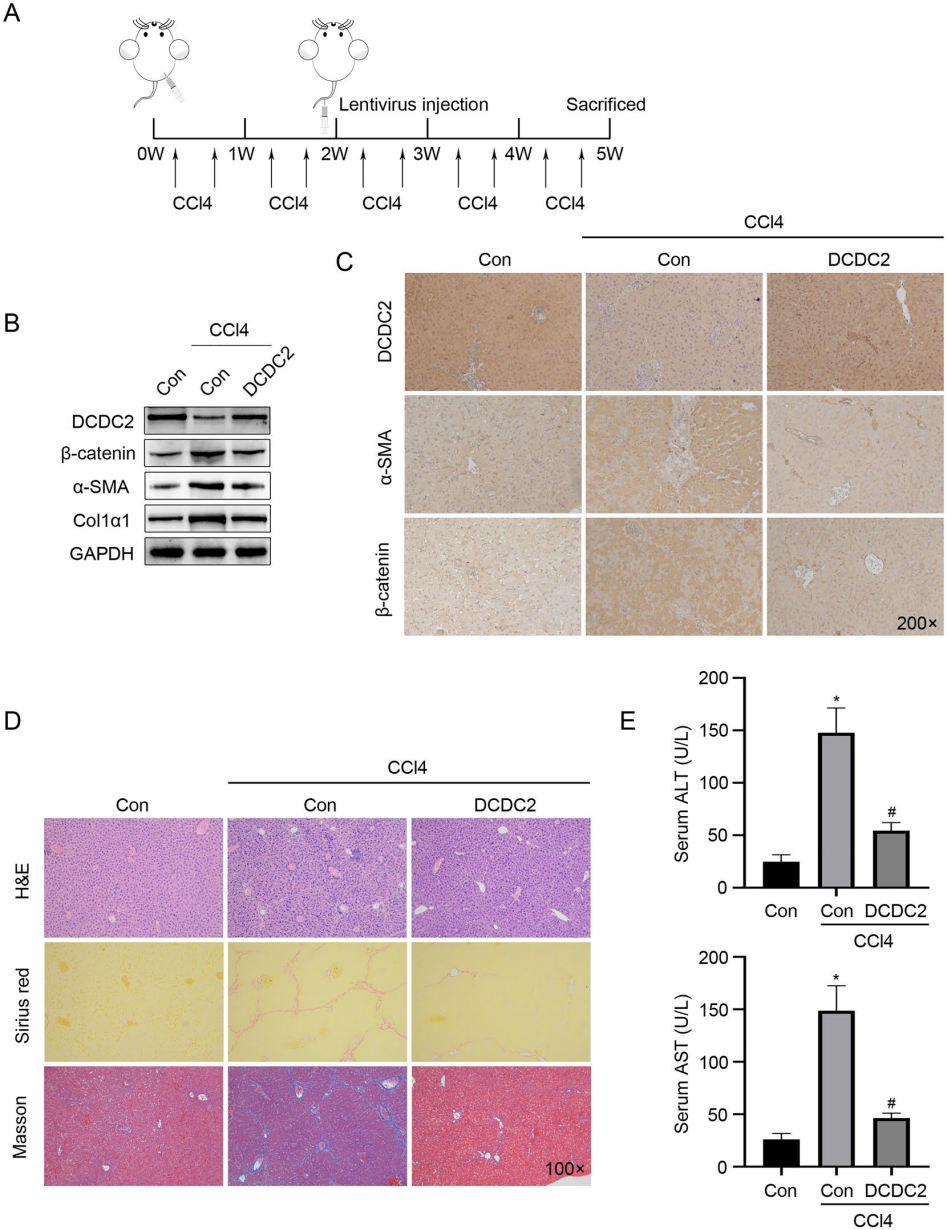
Overexpression of DCDC2 improves liver fibrosis in CCl_4_-induced mouse model. (**A**) The timeline of the animal experiment. Two weeks following the initial CCl_4_ injection, a single dose of lentivirus carrying DCDC2 or the control was administered via the tail vein. After 5 weeks of CCl_4_ treatment, the mice were sacrificed. (**B**) The protein expression levels of β-catenin, α-SMA, and Col1α1 were detected by western blot after overexpression of DCDC2 in mice. (**C**) Immunohistochemistry was performed to detect the expression of β-catenin and α-SMA in tissues from mice treated with either control virus or LV-DCDC2. (**D**) H&E, Sirius red staining and Masson trichrome staining in CCl_4_ mice following LV-DCDC2 treatment. (**E**) Serum ALT and AST levels were determined in CCl_4_ mice after LV-DCDC2 treatment. The experiment was repeated at least three times and statistical data were presented as mean ± SD. * compare with Con group, # compare with CCl_4_ group, *, # *P* < 0.05.

The in vitro experimental outcomes suggested an anti-fibrosis effect of DCDC2. To confirm these findings in vivo, mice were treated with CCl_4_ to induce liver fibrosis concurrently with DCDC2 overexpression (Fig. 5A). Subsequently, western blot analysis revealed reductions in the levels of α-SMA and β-catenin (Fig. 5B). These results were further confirmed through immunohistochemistry staining (Fig. 5C). Additionally, liver sections underwent hematoxylin and eosin, Sirius red staining, and Masson staining. The results indicated that the overexpression of DCDC2 alleviated CCl_4_-induced fibrous tissue deposition, liver injury, and fibrosis (Fig. 5D). Furthermore, a consistent decrease in the levels of alanine aminotransferase (ALT) and aspartate aminotransferase (AST) was observed in the serum from LV-DCDC2-treated groups (Fig. 5E). These data indicated that DCDC2 possessed the ability to inhibit collagen fiber deposition and liver fibrosis in vivo.

## Discussion

Liver fibrosis represents a crucial pathological progression in the early stage of various liver conditions including hepatic cirrhosis and liver cancer^34,35^. HSC emerge as the principal collagen-synthesizing cells responsible for the abnormal increase of the ECM, thereby playing a key role in liver fibrogenesis^36,37^. Activated HSC undergo subsequent transdifferentiation into myofibroblast-like cells, which exhibit enhanced proliferation and fibrogenic phenotype^38^. Although mild liver fibrosis is deemed reversible, the current absence of effective antifibrotic drugs or approved therapies underscores the critical need to investigate the mechanism underlying liver fibrosis^39^. In recent years, EMT has emerged as one of the mechanisms by which myofibroblasts are produced in liver fibrosis^40,41^. HSC exhibit high plasticity, demonstrating the ability to undergo EMT and mesenchymal-epithelial transition (MET)-like processes. This plasticity is sustained by their ability to express both epithelial and mesenchymal markers and to differentiate into myofibroblasts under certain conditions. These transitions are regulated by pathways activated during liver injury, highlighting the potential of HSC in liver regeneration and repair^32,33,42,43^. Thus, inhibiting HSC activation, proliferation and EMT-like processes may be a functional mechanism against liver fibrosis. Recently, emerging evidence suggested that the Wnt/β-catenin signaling pathway is associated with the development of liver fibrosis, rendering it a primary therapeutic target for treating this condition^44,45^. Therefore, investigating the mechanism of the Wnt/β-catenin signaling pathway for HSC activation is of crucial significance for the treatment of liver fibrosis.

DCDC2, a member of the DCX family, has been identified as a vital candidate susceptibility gene for dyslexia. The brain-specific protein is involved in neuronal cell migration and interacts with microtubules^46,47^. Mutations in the *DCDC2* gene may induce dyslectic reading disabilities owing to abnormal neuronal migration^48^. Additionally, Markus Schueler et al. confirmed that mutations in DCDC2 can lead to renal-hepatic ciliopathy by disrupting Wnt signaling^28^. Additionally, recent studies have increasingly demonstrated the involvement of DCDC2 in the occurrence and development of various malignant tumors, including ovarian cancer, breast cancer, prostate cancer, colorectal cancer, and HCC^27,49–52^. Notably, DCDC2 has been identified as a candidate tumor suppressor gene in HCC through triple combination array analysis. However, the specific role of DCDC2 in HSC activation and liver fibrosis remains unexplored.

To confirm the effect of DCDC2 in liver fibrosis, we collected human normal liver tissues and liver fibrosis tissues. Our analysis revealed a down-regulation of both RNA and protein levels of DCDC2 in the human liver fibrosis group, accompanied by increased levels of α-SMA and Col1α1. The findings were consistent in mice subjected to CCl_4_-induced liver fibrosis. Furthermore, we observed that DCDC2 co-localized with activated HSC and was downregulated in the activated HSC, which suggests a potential role of DCDC2 in HSC activation. To investigate the relationship between DCDC2 and HSC activation, we built an in vitro model utilizing TGF-β1 to induce HSC activation. Our findings revealed that α-SMA and Col1α1 expression levels were enhanced in a time- and dose-dependent manner following HSC activation, while DCDC2 exhibited the opposite trend. Additionally, we assessed the level of DCDC2 using an immunofluorescence assay, which indicated a decrease in DCDC2 expression upon HSC activation. Subsequent intervention was conducted using DCDC2 overexpression lentivirus and DCDC2-sgRNA to investigate the impact of DCDC2 on HSC activation. Overexpression of DCDC2 resulted in a significant decrease in α-SMA and Col1α1 expression levels in HSC, whereas knocking down DCDC2 resulted in opposite changes. Additionally, the expression level of β-catenin was affected by DCDC2 changes. Considering these results, we sought to determine whether DCDC2 contributed to HSC activation through the Wnt/β-catenin signaling pathway. We observed the changes in β-catenin and its downstream target genes, including cyclinD1 and c-myc, induced by DCDC2, could be reversed by β-catenin agonists or antagonists. Moreover, DCDC2 attenuated the nuclear translocation of β-catenin induced by TGF-β1. To further demonstrate the effect of DCDC2 on the function of HSC, we analyzed cell cycle progression and cell proliferation using flow cytometry and EdU assay, respectively. These results indicated that DCDC2 can arrest HSC in the G0/G1 phase and attenuate cell proliferation. EMT, a crucial process involving the gradual transdifferentiation of non-polar epithelial cells into mesenchymal cells, played a pivotal role in liver fibrosis. Notably, HSC can undergo an EMT-like process and transition into cells exhibiting a more mesenchymal phenotype. Further experiments suggested that DCDC2 significantly inhibited EMT-like processes. The data suggested that silencing DCDC2 promoted while overexpressing DCDC2 inhibited, the TGF-β1-induced activation of HSC in vitro.

Finally, in vivo experiments conducted on CCl_4_-induced liver fibrosis mice further confirmed the aforementioned results. Our findings demonstrated that DCDC2 could reduce the levels of α-SMA, Col1α1, and β-catenin induced by CCl_4_. Pathological examination revealed that DCDC2 exhibited protective effects against liver injury and fibrosis caused by CCl_4_. Additionally, following DCDC2 treatment, a significant reduction in the serum levels of ALT and AST induced by CCl_4_ was observed.

In conclusion, DCDC2 exhibited a significant downregulation in TGF-β1-activated HSC and CCl_4_-induced liver fibrosis in mice. The overexpression of DCDC2 inhibited TGF-β1-induced HSC activation partly through the Wnt/β-catenin signaling pathway, thereby suppressing HSC proliferation and EMT-like processes in vitro, and attenuating CCl_4_-induced fibrogenic changes in vivo. These findings highlight the potential utility of DCDC2 in the prevention and treatment of liver fibrosis, offering a novel approach for clinical practice in this field.

## Materials and methods

### Human liver specimens

Ten normal and sixteen human liver fibrosis samples were taken from the Affiliated Hospital of Nantong University. All specimens were obtained with the informed consent of each patient. The study protocol received approval from the Ethics Committee of the Affiliated Hospital of Nantong University. A portion of the liver specimens was used for quantitative real-time polymerase chain reaction (qPCR), while another portion was fixed in 4% paraformaldehyde, followed by subsequent processing and embedding in paraffin. The remaining samples were stored at − 80 °C.

### Mouse model of liver fibrosis

Male C57BL/6 J mice (8 weeks) were obtained from the Experimental Animal Center of Nantong University. All the animal experimental protocols were approved by the Animal Ethics Committee of Nantong University. The study is reported in accordance with ARRIVE guidelines. Mice were randomly divided into different groups (n = 6). The fibrosis groups of mice were injected intraperitoneally with 10 μL/g CCl_4_ (diluted 1:9 (v/v) in olive oil) twice a week for 6 weeks and the control groups were administered equivalent volumes of olive oil only. To overexpress DCDC2 in the liver, a single dose of 1 × 10^7^ TU LV-DCDC2 (Miaoling Biology, Wuhan, China) or control virus was intravenously injected into the tail vein two weeks after the first CCl_4_ injection. The mice were sacrificed under anesthesia three days after the last CCl_4_ injection. Blood samples were obtained for the analysis of alkaline transaminase (ALT) and aspartate transaminase (AST). Liver tissues were collected for the following experiments. At the end of the experiment, animals were sacrificed by cervical dislocation under deep anesthesia with 2% isoflurane.

### Cell culture and treatment

LX-2, a human immortalized HSC line, was cultivated in complete Dulbecco’s modified Eagle’s medium (DMEM), which contained 10% fetal bovine serum (Gibco, USA) and 1% penicillin–streptomycin (NCM Biotech, Suzhou, China) at 37 °C in 5% CO_2_/95% air. When the LX-2 cells’ density grew to 70–80%, 10 ng/ml TGF-β1 was used to stimulate the cells for 24 h to mimic the activation of HSC in vitro as a cellular model.

### Cell transfection

The lentiviral carrying DCDC2 overexpressing plasmid and small guide RNA (sgRNA) targeting DCDC2 (DCDC2-sgRNA) were provided by Miaoling Biology (Wuhan, China). The cells were plated in a 6-well plate at a density of 50–60% and were transiently transfected following the manufacturer's guidelines. The medium was replaced with complete DMEM 6 h after transfection. The cells were cultured for an additional 48 h and used for the following experiments.

### RNA isolation and qPCR

The total mRNA was extracted by RNA-Quick Purification Kit (Yishan Biotech, Shanghai, China). The HiScript III RT SuperMix for qPCR (Vazyme Biotech, Nanjing, China) was used to reverse transcribe the extracted total RNA into cDNA. The qPCR analysis was detected by a LightCycler96 (Roche) with SYBR (Servicebio Biotech, Wuhan, China). The expression levels of relative mRNA were obtained by the 2^−ΔΔCT^ method. The primer sequences of DCDC2 was (F:CCGTGCACTATCTTCTTGATTG; R:CTGATTCAAGGTTTTTCTGGGG).

### Western blot analysis

Liver tissues and cells were lysed with radioimmunoprecipitation assay (RIPA) buffer containing 1% protease and phosphatase inhibitor cocktail (NCM Biotech, Suzhou, China). The proteins were loaded to SDS-PAGE gels, and then transferred to polyvinylidene fluoride (PVDF) membrane (Roche Diagnostics Gmbh, Germany). After blocking by 5% nonfat milk, the membranes were incubated with the primary antibody against the following targets: DCDC2 (26,978–1-AP, Proteintech, Wuhan, China), α-SMA (ab124964, Abcam, England), Col1α1 (AF6524, Beyotime Biotech, Shanghai, China), β-catenin (GB12015, Servicebio, Wuhan, China), CylcinD1 (26,939–1-AP, Proteintech, Wuhan, China), c-myc (10,828–1-AP, Proteintech, Wuhan, China), Lamin A/C (10,298–1-AP, Proteintech, Wuhan, China), E-cadherin (20,874–1-AP, Proteintech, Wuhan, China), N-cadherin (22,018–1-AP, Proteintech, Wuhan, China), Vimentin (10,366–1-AP, Proteintech, Wuhan, China), Snail1 (13,099–1-AP, Proteintech, Wuhan, China) and GAPDH (60,004-1-Ig, Proteintech, Wuhan, China) overnight at 4 °C. After washing three times with Tris-buffered saline (TBS) supplemented with 1% Tween-20 (TBST), the membranes were incubated with secondary antibodies for 1 h at room temperature. The protein bands were visualized by a bioimaging system (Bio-Rad, USA) using the enhanced chemiluminescent kit (NCM Biotech, Suzhou, China). Quantitative analyses were performed by ImageJ software. The original blots are available in the supplementary material.

### Immunofluorescence staining

LX-2 cells were inoculated on 24-well plates and then fixed in 4% paraformaldehyde for 30 min. Liver tissue sections and cells were blocked with 5% bovine serum albumin (BSA) at room temperature for 1 h. Subsequently, primary antibodies were incubated overnight at 4 °C, followed by peroxidase-conjugated secondary antibodies at room temperature for 1 h in the dark. Finally, nuclei were stained with DAPI for 5 min under dark conditions. The fluorescence was captured using the inverted fluorescence microscopy.

### Immunohistochemistry staining

Liver tissue sections were immersed by endogenous peroxidase blocking for 10 min and blocked with 5% BSA for 1 h at room temperature. Primary antibodies were applied to the sections overnight at 4 °C. After three washes with PBS, the sections were incubated with HRP-conjugated secondary antibody at 37 °C for 20 min and diaminobenzidine (DAB) chromogen at room temperature for 5–8 min (PV-9000, ZSGB-BIO, Beijing, China). The nuclei were counterstained with hematoxylin. The sections were dehydrated in ascending series of ethanol, cleared in xylene, sealed with neutral gum and examined using Leica light microscope.

### EdU incorporation assay

In brief, according to the instructions of the BeyoClick EdU detection kit (Beyotime Biotech, Shanghai, China), LX-2 cells were incubated with a 10 μM concentration of EdU. The cell nuclei were labeled with Hoechst 33,342 and visualized using inverted fluorescence microscopy.

#### Cell cycle analysis

Cells were fixed in 70% cold ethanol overnight at 4 °C. According to the instructions of the cell cycle detection kit (Keygen Biotech, Nanjing, China), fixed cells were washed with PBS, and then incubated with the staining solution for 30 min. Flow cytometer (Beckman, USA) was used to assess the cell cycle and NovoExpress software (Agilent, USA) was used to analyze the data.

#### Histopathological analysis

Hematoxylin and eosin (H&E) staining was conducted to observe liver injury, while Sirius red and Masson trichrome staining were performed to assess collagen deposition.

#### Statistical analysis

All statistical analyses were conducted using GraphPad Prism 8.0, and the results were presented as the mean ± SD of at least three independent experiments. Statistical comparisons between the two groups were conducted using Student’s t-test. *P* < 0.05 was considered statistically significant.

### Supplementary Information

Supplementary Information.

## Data Availability

Data is provided within the manuscript or supplementary information files.
