# Supplementary material for: DCDC2 inhibits hepatic stellate cell activation and ameliorates CCl4-induced liver fibrosis by suppressing Wnt/β-catenin signaling
Source: Sci Rep. 2024 Apr 24;14:9425. doi: 10.1038/s41598-024-59698-w (PMC11043443; doi:10.1038/s41598-024-59698-w)

Original blots in Figure 1

Figure 1C

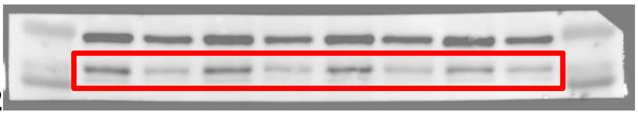

DCDC2

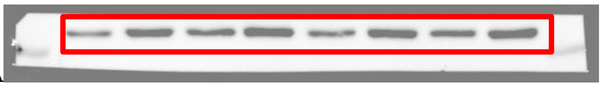

α-SMA

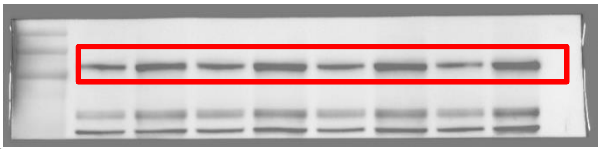

Col1α1

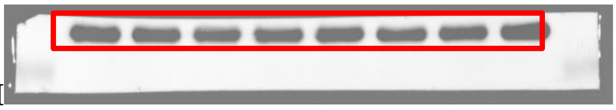

GAPDH

Figure 1E

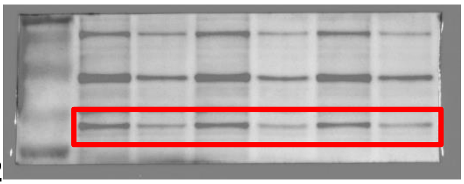

DCDC2

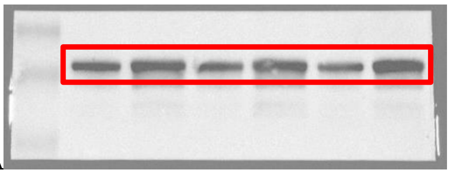

α-SMA

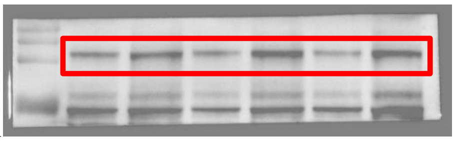

Col1α1

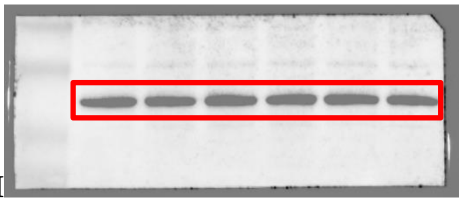

GAPDH

Original blots in Figure 2

Figure 2A

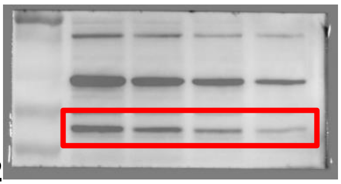

DCDC2

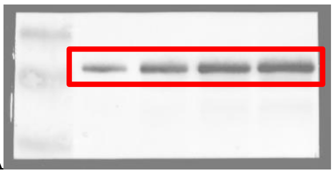

α-SMA

Col1α1

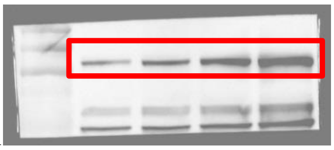

GAPDH

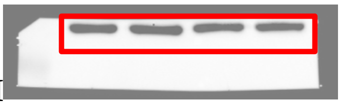

Figure 2B

DCDC2

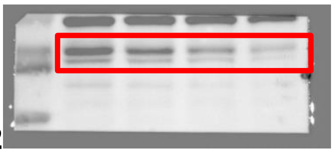

α-SMA

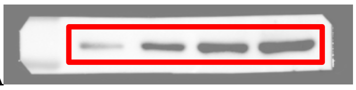

Col1α1

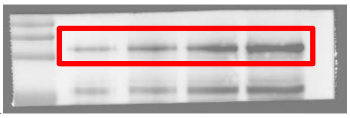

GAPDH

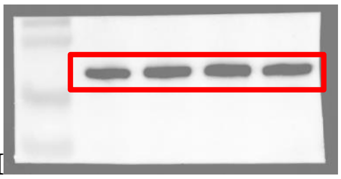

Figure 2E

DCDC2

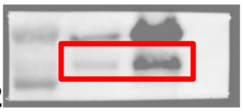

GAPDH

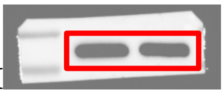

DCDC2

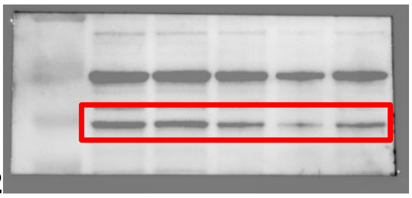

GAPDH

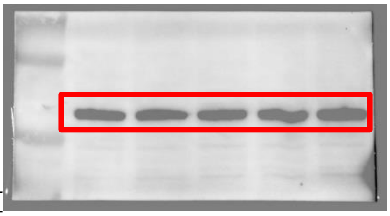

Original blots in Figure 3

Figure 3A

β-catenin

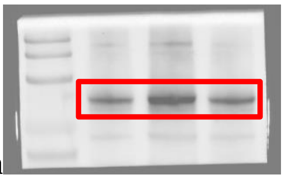

α-SMA

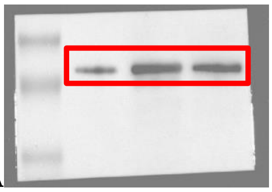

Col1α1

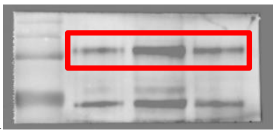

GAPDH

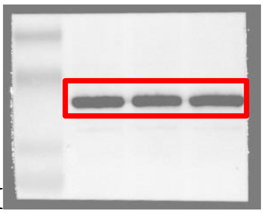

Figure 3B

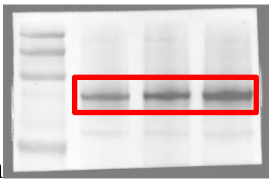
β-catenin

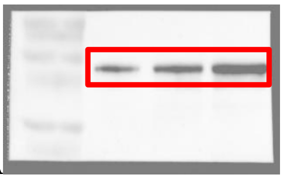
α-SMA

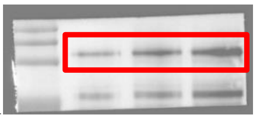
Col1α1

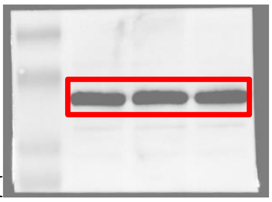
GAPDH

Figure 3C

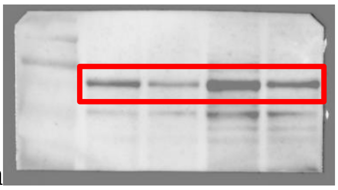
β-catenin

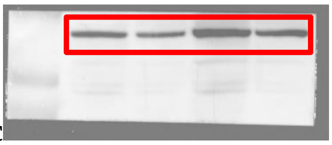
c-myc

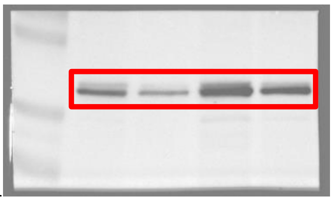
CyclinD1

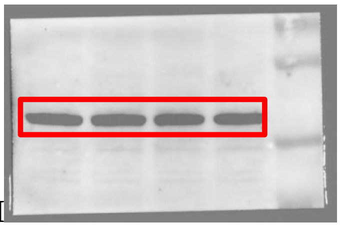
GAPDH

Figure 3D

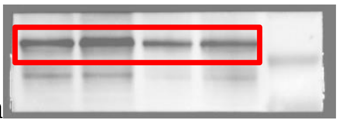
β-catenin

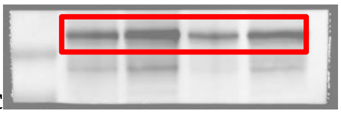
c-myc

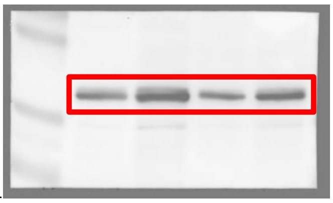
CyclinD1

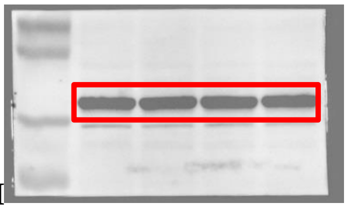
GAPDH

Figure 3E

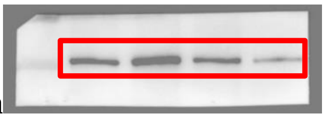
β-catenin

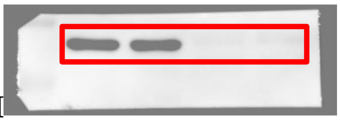
GAPDH

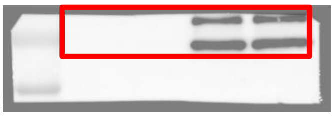
Lamin A/C

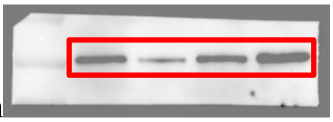
β-catenin

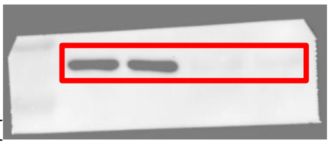
GAPDH

Lamin A/C
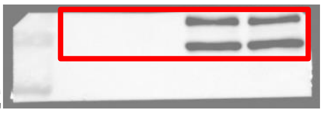

Original blots in Figure 4

Figure 4E

E-cadherin
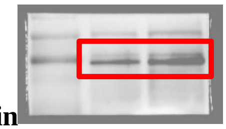

N-cadherin
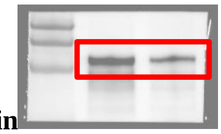

Vimentin
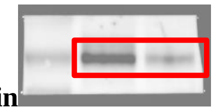

Snail
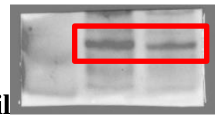

GAPDH
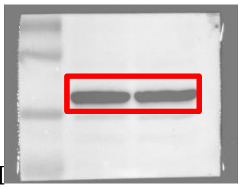

E-cadherin
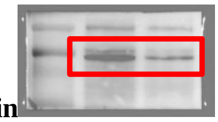

N-cadherin
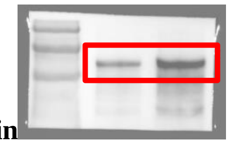

Vimentin
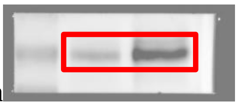

Snail
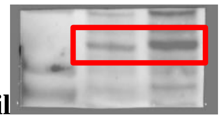

GAPDH
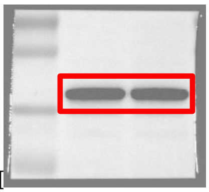

Original blots in Figure 5

Figure 5B

DCDC2
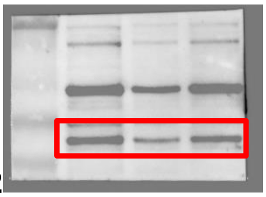

β-catenin
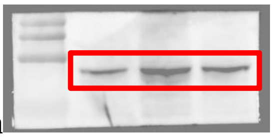

α-SMA
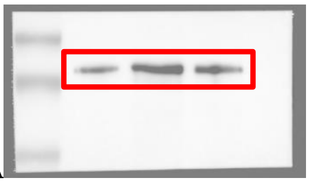

Col1α1
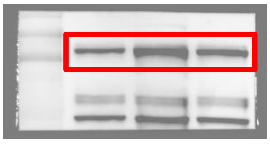

GAPDH
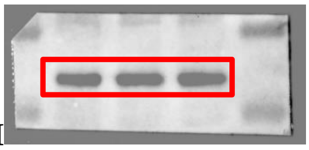

Supplement: Supplementary file 1 — Supplementary Information. [file 41598_2024_59698_MOESM1_ESM.pdf]
